# Full Hill-type muscle model of the I1/I3 retractor muscle complex in *Aplysia californica*

**DOI:** 10.1007/s00422-024-00990-3

**Published:** 2024-06-26

**Authors:** Ravesh Sukhnandan, Qianxue Chen, Jiayi Shen, Samantha Pao, Yu Huan, Gregory P. Sutton, Jeffrey P. Gill, Hillel J. Chiel, Victoria A. Webster-Wood

**Affiliations:** 1 Department of Mechanical Engineering, Carnegie Mellon University, 5000 Forbes Ave., Pittsburgh, PA 15213 USA; 2 Department of Biology, Case Western Reserve University, 2080 Adelbert Road, Cleveland, OH 44106-7080 USA; 3 Department of Nutrition, Case Western Reserve University, 2080 Adelbert Road, Cleveland, OH 44106-7080 USA; 4 Department of Neurosciences, Case Western Reserve University, 2080 Adelbert Road, Cleveland, OH 44106-7080 USA; 5 Department of Biomedical Engineering, Case Western Reserve University, 2080 Adelbert Road, Cleveland, OH 44106-7080 USA; 6 Department of Biomedical Engineering, Carnegie Mellon University, 5000 Forbes Ave., Pittsburgh, PA 15213 USA; 7 McGowan Institute for Regenerative Medicine, Carnegie Mellon University, 5000 Forbes Ave., Pittsburgh, PA 15213 USA; 8 School of Life and Environmental Sciences, University of Lincoln, Green Lane, Lincoln, LN67DL UK

**Keywords:** *Aplysia californica*, Hill-type model, muscle, dynamics

## Abstract

The coordination of complex behavior requires knowledge of both neural dynamics and the mechanics of the periphery. The feeding system of *Aplysia californica* is an excellent model for investigating questions in soft body systems’ neuromechanics because of its experimental tractability. Prior work has attempted to elucidate the mechanical properties of the periphery by using a Hill-type muscle model to characterize the force generation capabilities of the key protractor muscle responsible for moving *Aplysia*’s grasper anteriorly, the I2 muscle. However, the I1/I3 muscle, which is the main driver of retractions of *Aplysia*’s grasper, has not been characterized. Because of the importance of the musculature’s properties in generating functional behavior, understanding the properties of muscles like the I1/I3 complex may help to create more realistic simulations of the feeding behavior of *Aplysia*, which can aid in greater understanding of the neuromechanics of soft-bodied systems. To bridge this gap, in this work, the I1/I3 muscle complex was characterized using force-frequency, length-tension, and force-velocity experiments and showed that a Hill-type model can accurately predict its force-generation properties. Furthermore, the muscle’s peak isometric force and stiffness were found to exceed those of the I2 muscle, and these results were analyzed in the context of prior studies on the I1/I3 complex’s kinematics in vivo.

## Introduction

Understanding how neuromuscular systems coordinate complex multifunctional behavior requires a detailed understanding of both neural dynamics and the mechanics of the peripheral musculature. The periphery shapes the dynamics of each behavior differently due to changing mechanical advantages, contact interactions, and variations in the stiffness and damping of various muscle elements. For example, changing limb positions throughout walking changes the apparent stiffness at given joints (Silder et al. 2008). Similarly, variations in the shape of peripheral components can change the mechanical advantage of individual muscles to change overall forces in different behaviors (Olberding et al. 2019). Integral to such dynamic changes is the behavior and mechanics of individual muscles. These mechanics may vary as a function of species (McMahon 1984), muscle type (Srinivasan et al. 2007), fiber orientation (Kuthe and Uddanwadiker 2016) and degradation of the biomaterials that compose muscle tissue (Zhang et al. 2020). A detailed understanding of these properties is critical to understanding and modeling multifunctional behavior in neuromuscular systems.

Studying the neuromuscular control of soft-bodied animals and muscular hydrostatic systems, systems in which muscle plays both a structural and force-generating role such as tongues, trunks or tentacles (Longren et al. 2023), is particularly challenging due to the complex interactions of muscles throughout behavior. One model system for studying the neural control of a soft-bodied system is the sea slug, *Aplysia californica* (Webster-Wood et al. 2020). *Aplysia* generates multifunctional feeding behavior using a soft-bodied feeding structure made of muscle and cartilage. Feeding in *Aplysia* is typically classified into three primary behavioral types (Webster-Wood et al. 2020; Neustadter et al. 2007): (1) biting, which is an attempt to grasp food, (2) swallowing, which is an ingestive behavior in which seaweed is pulled into the esophagus by the feeding apparatus, and (3) rejection, which allows non-edible food to be pushed out of the feeding apparatus. Throughout each of these behaviors, the timing and degree of muscle activity vary, allowing all three behaviors to be generated with a limited number of neurons and a single periphery.

To understand how the nervous system coordinates the complex musculature of the *Aplysia* feeding apparatus to generate multifunctional behavior, researchers have used neuromechanical modeling (Sutton et al. 2004; Webster-Wood et al. 2020). Neuromechanical models allow hypotheses about functions of muscles and neurons to be generated and tested in simulations (Prilutsky et al. 2016; Valero-Cuevas and Santello 2017). However, for such models to be used to guide experimental research, they must be as biomimetic as possible while still enabling high throughput simulation (Webster-Wood et al. 2020). Models with varying bioplausibility have been developed for *Aplysia* feeding, which has greatly enhanced our understanding of multifunctional soft-bodied control in this system. These models range from conductance-based models without periphery or sensory feedback (Costa et al. 2020), to highly abstracted biomechanical models of key muscle forces and elastic elements (Sutton et al. 2004; Webster-Wood et al. 2020; Shaw et al. 2015) to complex kinematic models (Neustadter et al. 2007). Each of these models has provided insight into the neuromuscular system. However, detailed biomechanical models of the system are limited due to the dearth of muscle property data available in the literature. To date, few *Aplysia* muscles have been characterized in detail to facilitate modeling the force-frequency, length-tension, and force-velocity properties of each muscle. In fact, only the I2 protractor muscle has a detailed Hill-type model available as of the writing of this paper (Yu et al. 1999, 1997). The I2 muscle is a critical muscle for protracting *Aplysia*’s soft grasper called the odontophore during feeding (Hurwitz et al. 1996).

In this paper, we characterize and model the I1/I3 muscle complex, a muscle critical for retracting *Aplysia*’s odontophore during feeding (Lu et al. 2015). It is hypothesized that the basic protraction/retraction cycle of the feeding behavior is primarily driven by I1/I3 in conjunction with I2 (Sutton et al. 2004). To facilitate the creation of a Hill-type muscle model of the I1/I3 for use in biomechanical modeling, force-frequency, length-tension, and force-velocity experiments were conducted using a length-controlled servomotor. From these data, the series elasticity of the muscle was estimated, models were fit for each experimental condition, and the model was implemented in MATLAB. The model was further validated by simulating the muscle response and comparing the force to EMG data collected in semi-intact reduced I1/I3 preparations under the same stimulation conditions. The muscle model presented here adds to our understanding of the mechanics in the *Aplysia* feeding system and will allow researchers to improve the realism of future *Aplysia* neuromechanical models.

## Methods

**Fig. 1:**
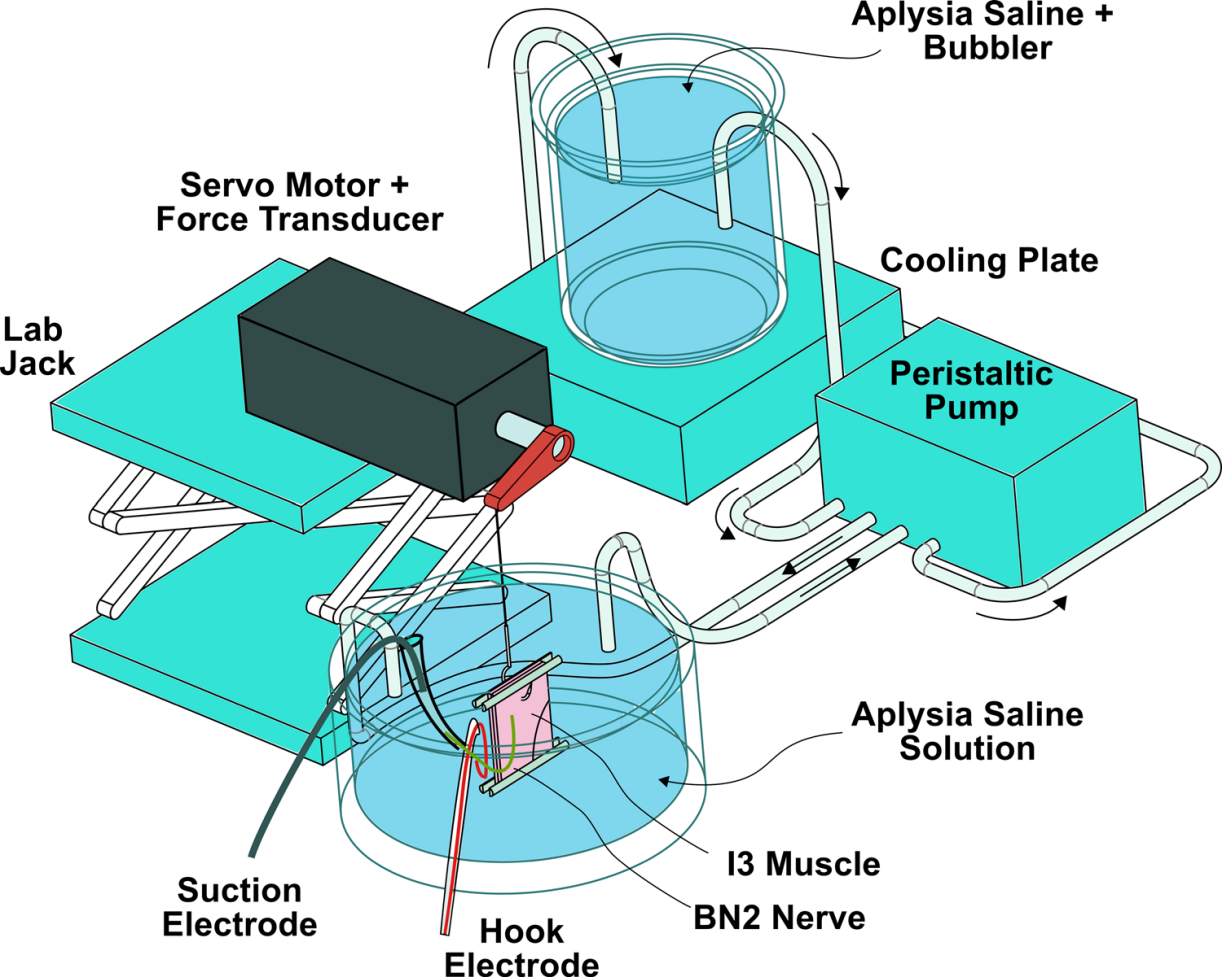
Schematic of the experimental muscle testing environment. The muscle is glued to the base of the testing dish, and a hook is used to affix the top side of the muscle to the servo motor for length control and force measurement. Hook and suction electrodes were glued to the nerve as described in Sect. 2.1. To accommodate differences in muscle size, the servo motor was mounted to a lab jack, which made it possible to set the initial position of the muscle to $L_0$. The muscle was maintained at a steady temperature with oxygenated *Aplysia* saline throughout testing

### Muscle and nerve preparation

*A. californica* of mass between 200 and 450 g were maintained in aquaria containing artificial seawater at a temperature of 16°C. Only *Aplysia* that exhibited biting cycles between 3–5 s of hand-fed nori were selected. The animal was then placed on a dissection tray and anesthetized with a 50% (vol./mass) injection of isotonic 333 mM MgCl$_2$ solution. The completion of anesthetization was confirmed by gently touching the animal’s rhinophores and verifying there was no movement.

After anesthetization, the left half of the I1/I3 muscle was isolated from the buccal mass. To expose the underlying buccal mass, incisions were made on the dorsal surface of the animal. The buccal mass was removed from the animal by cutting away connective tissue, cutting the esophagus, and severing the connection to the cerebral ganglion. The buccal mass was then weighed, and the mass was recorded. The diameter of the buccal mass, $D_{BM}$, was measured on the midline between the jaw and the lateral groove via a caliper. The intact length of the I3 muscle, $L_0$, was calculated by assuming the I1/I3 muscle complex forms an ideal cylinder with diameter $D_{BM}$, and that $L_0 = \pi D_{BM}/2$. Following measurements, buccal nerves 1 and 3, the radular nerve, and the esophageal nerves were severed bilaterally. Buccal nerve 2 (BN2), which carries the axons of identified motor neurons B3/B6/B9 and other neurons that innervate the I3 muscle, was left intact on the left side but severed on the right side.

Given the semi-rectangular shape of the isolated I1/I3 muscle complex, a mechanical interface is needed between the top edge of the muscle and the connection to the servomotor to ensure the contribution of force from the entire muscle is recorded. To provide a mechanical connection for the servo motor to pull against, a metal paperclip was inserted through the anterior portion of the jaws until it protruded through the esophagus. The paperclip was then tightly pressed against the I1/I3 complex using pliers. Once the paperclip was secured, a cut was made on the dorsal surface of the I3 muscle along the anteroposterior axis to separate the halves of the I1/I3 muscle.

To complete muscle isolation, the half of the I1/I3 muscle complex attached to the paper clip was isolated from the remaining buccal mass tissue. First, the odontophore was cut away from the I1/I3 complex. Second, the isolation of the I1/I3 complex was completed by cutting along the antero posterior axis on the ventral side of the muscle, and discarding the denervated half of the I1/I3 complex. Finally, the remaining esophageal tissue was cut away from the muscle.

With one-half of the I1/I3 complex isolated, it was then transferred to the testing dish. For testing, the I1/I3 muscle was secured to a 50 mm diameter PDMS-coated glass dish as follows: A 1.6 mm diameter wooden dowel was split in half with a safety razor. On the ventral side of the tissue, a single half of the split dowel was secured to one side of the I3 muscle using cyanoacrylate glue and sutures (Fig. 1). The remaining half of the dowel was glued on the opposite side of the muscle and aligned with the sutured dowel. The ventral side of the I1/I3 complex with the dowels was then glued to the bottom of the PDMS-coated dish with cyanoacrylate. Following gluing, the dish was continuously perfused with fresh saline via a peristaltic pump that was connected to a reservoir containing a bubbler for oxygenation (Fig. 1).

To provide length control and force measurements, the paperclipped end of the I1/I3 muscle was connected to the rotary lever of a servomotor (Aurora Scientific Dual Model Lever System 305B-LR). A bent suture needle was hooked under the metal paperclip, and the suture was tied to the arm of the servo motor. The servomotor’s height was adjusted via a laboratory jack until the I1/I3 muscle’s vertical height equaled $L_0$.

Electrodes were connected to the BN2 for stimulation and recording. First, to record activity in the BN2 in response to stimulation, a hook electrode was prepared according to the procedure outlined in Cullins et al. (2010), and secured to the intact BN2 near its midlength via cyanoacrylate glue. The hook electrode was connected to the amplifier (AM systems Differential Amplifier Model 1700, A-M Systems, Everett, WA) and monitored for spontaneous action potentials to ensure the electrode functionality. At this point in preparation, the BN2 connection to the buccal ganglia was severed and a suction electrode was connected to the free end of the nerve for stimulation. The suction electrode was prepared according to Lu et al. (2013). The electrode tip was cut such that the inner diameter of the electrode was slightly less than the diameter of the nerve to ensure a good seal. The suction electrode was positioned near the nerve end and a small suction force was applied to pull the nerve into the tip. The nerve was then glued to the electrode, and a chlorided silver wire (0.010 inch diameter) was inserted into the electrode such that a small gap was observed between the wire and the nerve.

### Stimulation

The muscle was activated via bi-phasic electrical stimulation of the BN2 with the suction electrode. The stimulus was a bi-phasic charge-balanced current waveform which has been found to have a reduced risk of induced tissue damage than monophasic pulses (van den Honert and Mortimer 1979). The bi-phasic pulse had a pulse width of 1 ms, with an interpulse delay of 1 ms before the start of the exponential charge balancing phase. To produce the bi-phasic current, the bi-phasic waveform was programmed in a function generator (Siglent SDG 1032x). The voltage output of the signal generator was converted to a scaled current output via a linear stimulator isolator (WPI A395). These parameters are the same as those used previously for the characterization of the *Aplysia* I2 protractor muscle (Yu et al. 1999). The hook electrode was used to stimulate the nerve with the same settings if the suction electrode failed.

To determine the current amplitude to be used for muscle characterization experiments, each preparation was stimulated across a range of increasing current amplitudes from 250 to 400 $\mu$A. Amplitudes were tested in 50 $\mu$A intervals for 5 s each. The minimum current that evoked uniform action potentials was used for subsequent protocols with that muscle preparation. The typical amplitude used in the experiments was 400 $\mu$A. The frequency of the stimulus train was set by the frequency of the pulses from the DAQ to the trigger input of the signal generator. The current from the linear stimulator isolator was sent to the suction electrode via a differential amplifier (AM Systems Differential Amplifier Model 1700).

### Servomotor and recording system

**Fig. 2:**
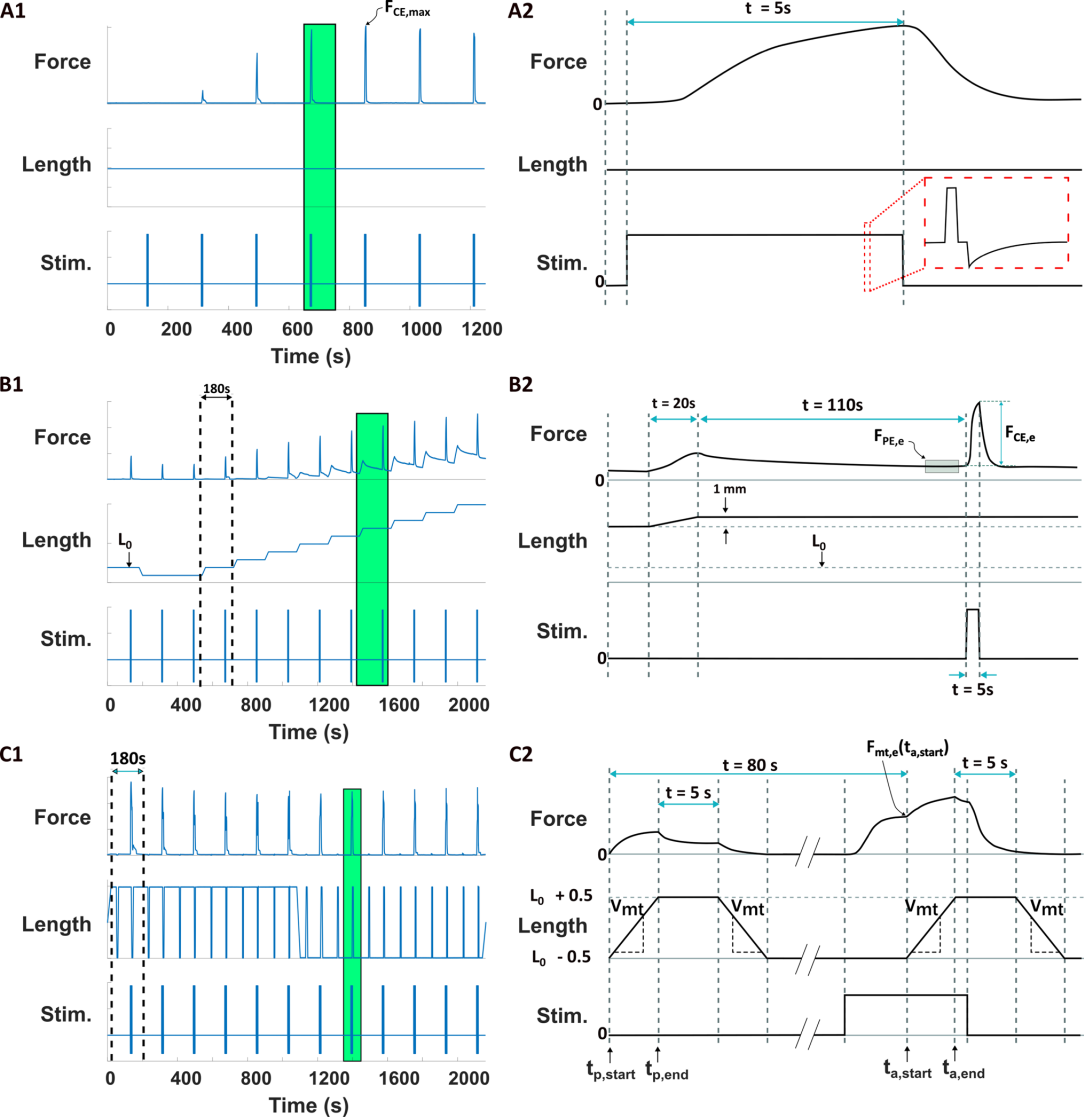
Force-frequency, length-tension and force-velocity protocols (left panels), with single episodes zoomed-in (right panels). Green box in left column indicates area that is zoomed in. **A1** Force frequency protocol. **A2** Muscle is kept at a constant length. Stimulation with a biphasic current pulse train is applied for 5 s (“Stim" plot). The biphasic current pulse used to stimulate the muscle is indicated in the inset. **B1** Length tension protocol. Muscle is ramped in 1 mm increments from the rest length to 8 mm past the rest length. Dashed lines indicate the start and end of a single episode. **B2** A single lengthening episode lasts 180 s in order to allow passive viscoelastic forces to decay. The estimate of the steady-state passive force, $F_{PE,e}$, and active force, $F_{CE,e}$ are indicated. Only episodes 3-12 were used to fit the length-tension relationship, which corresponded to $L_0 - 1$ mm to $L_0 + 8$ mm. **C1** Force velocity protocol. All shortening experiments are carried out first before the lengthening experiments. Dashed lines indicate the start and end of a single episode. **C2** Each episode consists of a first ramp where the muscle is not stimulated to estimate the passive viscoelastic forces, followed by a second ramp where the muscle is stimulated to determine the total force-velocity properties (active + passive forces). The isometric force at the start of the ramp, $F_{mt,e}(t_{a,start})$, and the start and end times of the second ramp, $t_{a,start}$ and $t_{a,end}$, are indicated

The control of the muscle’s effective length ($L_{mt}$) with time was accomplished with the use of a servomotor equipped with a 4 cm rotary lever arm (Aurora Scientific Dual Model Lever System 305B-LR ). The servomotor was capable of length changes of 20 mm and measuring forces from 0-10 N. The temporal sequence of length changes, stimulation and measurements of force and length (Fig. 2) was programmed in Axograph software and communicated to the servomotor and the stimulator via a data acquisition system (DAQ) (NI USB-6218). Force and length measurements from the servomotor controller and of the measured potentials from the hook electrode were recorded via Axograph at a sampling rate of 5 kHz. Measurements from the hook electrode were amplified with a differential amplifier (AM Systems Differential Amplifier Model 1700) prior to digitization. The amplifier was set to a low pass frequency of 300 Hz, a high pass frequency of 500 Hz and a gain of 10000.

### Experimental procedure

Experiments were performed as either pairs of *force-frequency & length-tension* or *force-velocity & length-tension* to minimize the total duration of the experiment to reduce muscle fatigue. A total of $n=5$ force-frequency, $n=5$ length-tension and $n=4$ force-velocity experiments were performed.

#### Force-frequency experiments

The force-frequency experiments were performed by isometric contraction of the muscle while it was held at the rest length ($L_0$) by the servomotor (Fig. 2.A1). The muscle was subjected to stimulation trains of frequencies ($f_{stim}$) from 2 to 38 Hz in 6 Hz increments. Each stimulation train lasted for 5 s (Fig. 2.A2). To minimize the effects of muscle fatigue, the start of a subsequent stimulation train occurred 180 s after the start of the prior train.

#### Length-tension experiments

Length-tension experiments were performed to determine both the passive (unstimulated) and active (stimulated) force generation properties of the muscle as a function of its length (Fig. 2.B1). Each length-tension data point was determined using isometric experiments where the length was controlled as the input and force was the measured output (Winters et al. 2000). The muscle was subjected to the following changes in length relative to the rest length ($L_0$): 0,-1,-1, 0, 1, 2, 3, 4, 5, 6, 7 and 8 mm. Note that negative changes in length indicate shortening of the muscle relative to its rest length, positive changes in length indicate lengthening of the muscle relative to its rest length and zero indicates the muscle was at the rest length. The muscle exhibited significant stress-relaxation effects when stretched. To minimize the effect of the stress-relaxation effects on the length-tension data, 180 s were allowed to elapse between each length change ramp (Fig. 2.B1). Length changes were applied using a linear ramp over 20 s to minimize damage to the tissue from stretching or contracting too quickly (Fig. 2.B2). Note this slow stretch and long wait period allow us to measure near steady-state passive forces but neglect transient passive dynamics (See Discussion). At each length, the muscle was stimulated at a frequency $\ge$ 26 Hz for 5 s using the current identified by the procedure outlined in Sect. 2.2. Stimulation was applied 110 s after the preceding length-change ramp to allow both passive and active force to be determined at each length (Fig. 2.B2). To determine the passive force, the measured force was averaged over the 10 s period prior to the onset of stimulation. The active force was found by averaging the measured force during the 1 s period centered around the peak force found during the stimulation period and subtracting the passive force.

#### Force-velocity experiments

Isokinetic experiments were performed to quantify the effect of shortening and lengthening velocities on force production and to estimate the series elastic stiffness of each muscle (Fig. 2.C1). Each trial had two phases: (1) passive force measurement and (2) active force measurement (Fig. 2.C2). For each lengthening trial, the muscle started at $L_0 - 0.5$ mm and lengthened at a constant velocity $V_{mt}$ until it reached $L_0 + 0.5$ mm. Similarly, for shortening trials, the muscle started at $L_0 + 0.5$ mm and shortened at a constant velocity $V_{mt}$ until it reached $L_0 - 0.5$ mm. For each velocity tested, the muscle underwent the passive force measurement protocol first. After this protocol, the muscle was returned to its starting length, allowed to rest until 80 s had elapsed from the start of the passive protocol, and then underwent the active force measurement protocol for the same velocity. Six lengthening velocities (0.25, 0.5, 1, 2, 4, 8 mm/s) and six shortening velocities ($-0.25, -0.5, -1, -2, -4, -8$ mm/s) were tested for each muscle. This velocity range was chosen to minimize the possibility of damaging the muscle from higher velocity movements. To minimize potential damage to the muscle and allow transient stress relaxation to converge, each trial was separated by 180 s.

*Passive force measurement * To measure the passive force for each velocity profile, length changes were first performed without stimulation. For a trial of a given velocity, $V_{mt}$, the muscle started at $L_0 + 0.5$mm, for shortening trials, or $L_0 - 0.5$mm, for lengthening trials, and then the length was changed at a constant velocity $V_{mt}$ until it reached $L_0 - 0.5$mm, for shortening, or $L_0 + 0.5$mm, for lengthening. The muscle was kept at this length for 5 s before returning to the starting length (i.e., $L_0 + 0.5$mm for shortening trials and $L_0 - 0.5$mm for lengthening trials).

*Active force measurement * After the appropriate passive force measurement for each velocity profile, active force measurements during length changes with stimulation were performed. For each velocity trial, 80 s after the start of passive force protocol had elapsed, the muscle was stimulated at a frequency $\ge$ 26 Hz for 10 s. After 5 s of stimulation, the force was measured as an estimate of the isometric force for that muscle. The muscle was then shortened or lengthened, as described for passive force measurements, while being stimulated. To minimize the effect of changes in the measured force caused by the movement of the nerve and the muscle, the force at a given velocity was normalized by the isometric force measured for that trial.

### Muscle model

A Hill-type muscle model composed of a series elastic element (SEE) that is in series with the contractile element (CE) and which are both parallel to a second elastic element (PE) (Winters et al. 2000; Zajac 1989) was used to model the I1/I3 muscle complex. In this model, the total force exerted by the muscle is given by Eq. 1, where $F_{mt}$ is the total muscle force, $F_{PE}$ is the force produced by the parallel elastic element, $F_{SEE}$ is the force produced by the series elastic element and $F_{CE}$ is the active force produced by the contractile element. $F_{PE}$ is a function of the total muscle length ($L_{mt}$). $F_{SEE}$ is a function of the “tendon" length $L_T$; note that there is not a physical tendon that exists in the I1/I3 complex, but rather, this is used to model internal connective tissue and structures like titin and the Z-band in sarcomeres that are suspected of being in series with the CE (Herzog et al. 2013). $F_{CE}$ is a function of the activation (*a*), the peak active isometric force ($F_{mto}$), length of the CE ($L_m$) and the velocity of the CE ($V_{m} = \frac{d L_m}{dt}$). Note that the velocity of the contractile element may be different from the velocity of the entire muscle complex, $V_{mt}$. Using this model, $F_{mt}$ can be expressed as:

1 $$\begin{aligned}&F_{mt} = F_{PE}(L_{mt}) + F_{CE}(a,F_{mto}, L_m, V_m) \end{aligned}$$

where:

2 $$\begin{aligned}&F_{CE}(a,F_{mto}, L_m, V_m) = F_{SEE}(L_{T}) \end{aligned}$$

To facilitate comparison with other muscles, Eq. 1 is typically normalized (Zajac 1989). A normalized version of Eq. 1 is produced by normalizing forces by $F_{mto}$, and lengths and velocities by $L_{mto}$, i.e., the length at which the peak active force, $F_{CE}$, occurs. ${\tilde{f}}_{mt}$, ${\tilde{f}}_{PE}$, ${\tilde{f}}_{SEE}$ and ${\tilde{f}}_{CE}$ represent the normalized $F_{mt}$, $F_{PE}$, $F_{SEE}$ and $F_{CE}$, respectively. ${\tilde{l}}_{mt}$, ${\tilde{l}}_{T}$, ${\tilde{l}}_{m}$, ${\tilde{v}}_{mt}$ and ${\tilde{v}}_{m}$ represent the normalized $L_{mt}$, $L_{T}$, $L_{m}$, $V_{mt}$ and $V_m$, respectively. The relationship between $F_{mt}$ and its normalized counterpart is given by:

3 $$\begin{aligned}&F_{mt} = F_{mto}\times {\tilde{f}}_{mt} \nonumber \\&F_{mt} = F_{mto} \times \left( {\tilde{f}}_{PE}\left( \frac{L_{mt}}{L_{mto}}\right) \right. \nonumber \\&\left. + {\tilde{f}}_{CE}\left( a, \frac{L_m}{L_{mto}},\frac{V_{m}}{L_{mto}}\right) \right) \nonumber \\&F_{mt} = F_{mto}\times \left( {\tilde{f}}_{PE}({\tilde{l}}_{mt}) + {\tilde{f}}_{CE}(a,{\tilde{l}}_{m},{\tilde{v}}_{m}) \right) \end{aligned}$$

where:

4 $$\begin{aligned}&{\tilde{f}}_{CE}\left( a, {\tilde{l}}_m, {\tilde{v}}_m\right) = {\tilde{f}}_{SEE}\left( {\tilde{l}}_T\right) \end{aligned}$$

The active muscle force dynamics is produced from the neurally-controlled activation of the contractile element, i.e., ${\tilde{f}}_{CE}$, which is a function of time (*t*) and the CE length and velocity (${\tilde{l}_m}$ and ${\tilde{v}_m}$, respectively). This is modeled using the form described by Zajac et al. (Zajac 1989) as the product of the activation dynamics (*a*(*t*)), length-tension properties (*LT*) and force-velocity (*FV*) properties as:

5 $$\begin{aligned}&{\tilde{f}}_{CE}(t) = a(t)\times FV({\tilde{v}}_{m}) \times LT({\tilde{l}}_{m}) \end{aligned}$$

The series elastic element is assumed to behave like a linear spring with a normalized spring constant $K_t$. Note that ${\tilde{l}}_t = {\tilde{l}}_{mt} - {\tilde{l}}_{m}$ is the length of the SEE. The slack length is unknown and hence set to 0, as was done by Yu et al. for the I2 muscle (Yu et al. 1999). ${\tilde{f}}_{SEE}$ can be calculated as:

6 $$\begin{aligned} {\tilde{f}}_{SEE} = K_t \cdot \tilde{l_t} \end{aligned}$$

### Data fitting

In the following sections, values determined from experiments are denoted by the subscript *e*. For instance, $F_{PE,e}$ represents the experimentally determined passive force, $F_{MT,e}$ represents the experimentally measured total force and so on.

#### Force-frequency

For each muscle tested, the baseline passive force was found by taking the average of the force in the interval 5 to 20 s before the start of stimulation. The active force ($F_{CE,e}$) was found from subtracting this baseline passive force from the total force ($F_{mt,e}$) during the 5-second stimulation period (Fig. 2.A). To determine the force at a given frequency of stimulation, $f_{stim}$, the $F_{CE,e}$ is averaged in a 2-second period centered at the time the peak $F_{CE,e}$ occurs during stimulation. The force was normalized by the maximum $F_{CE,e}$ of the force-frequency trials for a given dataset ($F_{CE,max}$). A sigmoid was used to capture the force-frequency relationship, $u_f$ (Roszek et al. 1994). This was fit from the data using the least-squares method fit in MATLAB such that:

7 $$\begin{aligned}&u_{f}(f_{stim}) = \frac{F_{CE,e}(f_{stim})}{F_{CE,max}} \nonumber \\&u_{f}({f_{stim}},{\textbf {A}}) = \frac{A_1}{A_1+A_2\cdot e^{-A_3\cdot (f_{stim} - A_4)}} \end{aligned}$$

where ${\textbf {A}} = \langle A_1, A_2, A_3, A_4 \rangle$ are coefficients determined during fitting.

#### Length-tension

At a given length of the muscle, $L_{mt}$, the passive force ($F_{PE,e}$) was extracted by averaging the force in the interval 2 to 10 s before the start of stimulation. Note that this passive force is assumed to be the same as that of the parallel elastic element because no force is developed in the CE when it is not active, and hence no force is developed in the SEE spring. The active force estimate, $F_{CE,e}$, is extracted at this length of the muscle complex by averaging the total force measured by the servomotor system in a 1-second interval centered around the peak force found during stimulation and then subtracting the passive force $F_{PE,e}$. Both $F_{CE,e}$ and $F_{PE,e}$ are normalized by the peak active force independently for each muscle, $F_{mto,e}$ to calculate $LT_e$ and ${\tilde{f}}_{PE,e}$, respectively. The length at which $F_{mto,e}$ occurs is used to determine the optimal muscle length $L_{mto,e}$, for each muscle individually. $L_{mt}$ is normalized by $L_{mto,e}$ to compute ${\tilde{l}}_{mt}$.

The normalized active force-length data is then fitted by a cubic polynomial (Roszek et al. 1994) using MATLAB’s lsqlin() routine for least-squares fitting as:

8 $$\begin{aligned} LT_{fit}\left( {\tilde{l}}_{mt},{\textbf {B}}\right) = \left( B_1\cdot {\tilde{l}}_{mt}^3 + B_2\cdot {\tilde{l}}_{mt}^2 + B_3\cdot {\tilde{l}}_{mt} + B_4\right) \nonumber \\ \end{aligned}$$

To find **B**, the following optimization was performed:

9 $$\begin{aligned} \min _{{\textbf {B}}} \quad&\sum _{i=1}^{N}(LT_{e}({\tilde{l}}_{mt,i})-LT_{fit}({\tilde{l}}_{mt,i},{\textbf {B}}))^2 \end{aligned}$$

10 $$\begin{aligned} \text {s.t.} \quad&\left( \sum _{i=1}^{4} B_i\right) -1=0 \end{aligned}$$

11 $$\begin{aligned}&3B_1+2B_2+B_3 = 0 \end{aligned}$$

12 $$\begin{aligned}&6B_1\cdot min({\tilde{l}}_{mt}) + 2B_2 \le 0 \end{aligned}$$

13 $$\begin{aligned}&6B_1\cdot max({\tilde{l}}_{mt}) + 2B_2 \le 0 \end{aligned}$$

Note that the constraints were placed so that the Hessian is negative definite and hence concave downward for the range of lengths tested, i.e.,$[min({\tilde{l}}_{mt}),max({\tilde{l}}_{mt})]$, and so that the stationary point of the cubic passes through [1, 1], i.e.,where the normalized $F_{mto,e}$ is located. ${\textbf{B}}=\langle B_1,B_2,B_3,B_4\rangle$ are coefficients, and *N* is the number of ${\tilde{l}}_{mt}$ datapoints.

To account for the series elasticity, ${\tilde{l}}_{m}$ is computed from ${\tilde{l}}_{mt}$ by:

14 $$\begin{aligned} {\tilde{l}}_{m} = {\tilde{l}}_{mt} - \frac{F_{CE,e}}{K_t} \end{aligned}$$

The active length-tension data, $F_{CE,e}$, is then re-fitted to ${\tilde{l}}_{m}$ using a cubic as was done in Eq. 8:

15 $$\begin{aligned} LT_{fit,m}({\tilde{l}}_{m},{\textbf {Y}}) = (Y_1\cdot {\tilde{l}}_{m}^3 + Y_2\cdot {\tilde{l}}_{m}^2 + Y_3\cdot {\tilde{l}}_{m} + Y_4) \end{aligned}$$

The normalized passive force-length data was fitted by an exponential equation using the fit() function in MATLAB as:

16 $$\begin{aligned} f_{PE,fit} = C_1 + C_2\cdot exp\left( C_3{\tilde{l}}_{mt}-C_4\right) \end{aligned}$$

where ${\textbf{C}}=\langle C_1,C_2,C_3,C_4\rangle$ represents the vector of coefficients.

#### Force-velocity

The force-velocity relationship at a given ramp velocity, $V_{mt}$, *FV* can be computed from the measured data ($F_{mt,e}$ etc.) by rearranging Eq. 3 and 5, such that:

17 $$\begin{aligned} FV({\tilde{v}}_m) = \frac{F_{mt,e}(t_{a,end}) - F_{PE,e}(t_{p,end})}{F_{mto,e} \cdot a \cdot LT(L_{mt}(t_{a,end}))} \end{aligned}$$

where $t_{p,start}$ and $t_{p,end}$ are the start and end times of the initial ramp (i.e.,the ramp without stimulation of the muscle). $F_{PE,e}$ is the passive force estimate, i.e.,$F_{mt,e}$ in the interval $[t_{p,start},t_{p,end}]$, and $t_{a,start}$ and $t_{a,end}$ are the start and end times of the second ramp (i.e.,the ramp with stimulation of the muscle). $F_{mt,e}(t_{a,end}) - F_{PE,e}(t_{p,end})$ is an estimate of the active force produced by the contractile element, $F_{CE}$ from Eq. 1. Both the passive and total forces at the end of the ramps, $F_{PE,e}(t_{p,end})$ and $F_{mt,e}(t_{a,end})$, respectively, are calculated by averaging the force data within a 1 ms interval centered at the time of interest for each estimate (i.e.,$t_{p,end}$ and $t_{a,end}$).

To reduce variability caused by the movement of the stimulating electrode when the muscle changes length (Yu et al. 1999), $F_{mto,e}$ in Eq. 17 is replaced with the maximum isometric force from the stimulation of the muscle ($F_{iso,est}^{max}$) just prior to the start of the second ramp. $F_{mt,e}(t_{a,start})$ is the experimentally measured isometric force (Fig. 2.C2). However, this force is measured at the start of the ramp, $L_{mt}(t_{a,start})$, which may not be $L_{mto}$, i.e.,the location where the maximum isometric force occurs. To compute $F_{iso,est}^{max}$ for this velocity episode, the active length-tension fit found in the length-tension experiments Eq. 8 was used to estimate the corrected isometric force, evaluated at ${\tilde{l}}_{mt,start} = \frac{L_{mt}(t_{a,start})}{L_{mto}}$:

18 $$\begin{aligned} F_{iso,est}^{max} = \frac{F_{mt,e}(t_{a,start})-F_{PE,e}(t_{p,start})}{LT_{fit}\left( {\tilde{l}}_{mt,start},{\textbf{B}}\right) } \end{aligned}$$

To get an estimate of the velocity of contraction of the contractile element (${\tilde{v}}_m$),$V_{mt}$ was transformed to ${\tilde{v}}_m$ by differentiation of the combined equations Eq. 3 and Eq. 6, and accounting for the series elasticity $K_t$ as follows:

19 $$\begin{aligned} \begin{aligned} {\tilde{v}}_{m} =&\left( \frac{V_{mt}(t_{a,end})}{L_{mto}} - \right. \\&\frac{1}{K_t \cdot F_{iso,est}^{max}} \cdot \\&\left. \frac{d(F_{mt,e}(t_{a,end})-F_{PE,e}(t_{p,end}))}{dt}\biggr |_{t=t_{a,end}} \right) \end{aligned} \end{aligned}$$

where $\frac{d(F_{mt,e}-F_{PE,e})}{dt}$, which represents the slope of the active force rise, is evaluated at $t_{a,end}$, and is calculated using the method outlined in the Series elasticity calculation (2.6.4).

The relationship between ${\tilde{v}}_{m}$ and *FV* is captured by a pair of piecewise hyperbolic functions of the form:

20 $$\begin{aligned} FV(\tilde{v}_{m}, {\textbf {D}}) = {\left\{ \begin{array}{ll} 1+\frac{D_1}{1+\frac{D_2}{{\tilde{v}}_{m}}} &{} \text {if } {\tilde{v}}_{m} \ge 0\\ \\ 1+\frac{D_3}{1-\frac{D_4}{{\tilde{v}}_{m}}} &{} \text {if } {\tilde{v}}_{m} < 0 \end{array}\right. } \end{aligned}$$

where ${\tilde{v}}_{m} \ge 0$ represents shortening and ${\tilde{v}}_{m} < 0$ represents lengthening. ${\textbf {D}}$ are coefficients of the fitted functions. MATLAB’s fit() function was used to perform least squares fitting.

To simulate the dynamics of the muscle, the *FV* relationship is inverted to yield the muscle velocity as a function of force. This inverse force-velocity relationship, *IFV*, was fitted using a double exponential function via MATLAB’s fit() function:

21 $$\begin{aligned} {\tilde{v}}_{m} = -E_1\cdot e^{E_2\cdot (FV - E_3)} + E_4\cdot e^{E_5\cdot FV} \end{aligned}$$

where ${\textbf {E}}$ represents the coefficients of the fit.

#### Series elasticity estimation

In isotonic quick-release experiments, the series elasticity, $K_t$, can be estimated by finding the slope of the force-length curve immediately after release. From the isokinetic experiments employed to obtain the force-velocity relationship, $K_t$ was estimated. It was important to estimate the series elasticity because Yu et al. found that its presence slowed the isometric force generation (Yu et al. 1999). First, combining Eqs. 1 and 2, yields:

22 $$\begin{aligned} F_{CE}(L_m, a , F_{mto},L_m, V_m)&= F_{SEE}(L_t) \nonumber \\&= F_{mt} - F_{PE}(L_{mt}) \end{aligned}$$

which is a relationship between the total force measured by the servomotor when the muscle is stimulated, $F_{mt}$, the passive elastic force, $F_{PE}$ which is measured during the unstimulated ramp of the force-velocity experiment, and the force produced by the series elastic element, $F_{SEE}$. The relationship between the length of the series elastic element, $L_t$ and $F_{SEE}$ is denoted by $\psi (L_t)$:

23 $$\begin{aligned} F_{SEE}&= \psi (L_t) = \psi (L_{mt} - L_{m}) \end{aligned}$$

Combining Eqs. 22 and 23, and differentiating with respect to time:

24 $$\begin{aligned} \frac{d(F_{mt}-F_{PE})}{dt} = \frac{d(\psi (L_{mt} - L_m))}{dt} \end{aligned}$$

Via the chain rule, this can be expanded further:

25 $$\begin{aligned} \frac{d(F_{CE})}{dt}&= \frac{d(F_{mt}-F_{PE})}{dt} \nonumber \\ \frac{d(F_{CE})}{dt}&= \frac{d(\psi (L_{mt} - L_m))}{d(L_{mt}-L_m)}\cdot \frac{d(L_{mt} - L_m)}{dt} \nonumber \\ \frac{d(F_{CE})}{dt}&= \frac{d(\psi (L_{mt} - L_m))}{d(L_{mt}-L_m)}\cdot (V_{mt} - V_m) \end{aligned}$$

Eq. 25 implies that at a given muscle length $L_{mt}$, the rate of change of the active force, $F_{CE} = F_{mt}-F_{PE}$, is linearly related to the velocity of the muscle, $V_{mt}$, with the slope $d(\psi (L_{mt} - L_m))/d(L_{mt}-L_m)$ equal to the stiffness, $K_t(L_{mt})$.

If the relationship between the series elastic force and the length of the muscle is linear, i.e.,$F_{SEE} = K_t \cdot (L_{mt} - L_m)$, then $d(\psi (L_{mt} - L_m))/d(L_{mt}-L_m) = K_t$. This relationship implies that $K_t$ is independent of $L_{mt}$. Eq. 25 then simplifies into:

26 $$\begin{aligned} \frac{d(F_{CE})}{dt} = K_t \cdot (V_{mt} - V_m) \end{aligned}$$

$K_t$ can then be scaled by:

27 $$\begin{aligned} K_t = \frac{L_{mto}}{V_{mt} - V_m} \cdot \frac{1}{F_{mto}} \cdot {\frac{d(F_{CE})}{dt}} \end{aligned}$$

$dF_{CE}/dt|_{t_a}$, evaluated at some time $t_a$ during the second force-velocity ramp (velocity $V_{mt}$, muscle is actively stimulated) is computed as follows. First, $F_{CE}(t_a)$ is calculated by subtracting the passive force during the first ramp as it passes through the muscle length of interest, $L_{mt}$, from the total force during the second ramp at that same length. This can be written as $F_{CE}(t_a) = F_{mt,e}(L_{mt}) - F_{PE,e}(L_{mt})$. This is repeated for all datapoints in the time interval spanning the ramp change in length of the muscle while it is actively stimulated $[t_{a,start},t_{a,end}]$. A fourth-order polynomial is fit to the $F_{CE}$ within this interval:

28 $$\begin{aligned} F_{CE,fit} = G_1t^4 + G_2t^3 +G_3t^2 + G_4t +G_5 \end{aligned}$$

where ${\textbf {G}}$ are fit coefficients.

The expression for the derivative is evaluated at $t_a$ to facilitate calculation of $K_t$ (Eq. 26):

29 $$\begin{aligned} \frac{d(F_{CE,fit})}{dt}\biggr |_{t_a} = 4G_1t_{a}^3 + 3G_2t_{a}^2 + 2G_3t_{a} + G_4 \end{aligned}$$

To evaluate Eq. 19, Eq. 29 is evaluated at the end of the second force velocity ramp, $t_{a,end}$.

#### Activation dynamics

The activation dynamics are modeled as a first-order differential equation described in Zajac (1989):

30 $$\begin{aligned} \frac{da'(t)}{dt}&= \frac{1}{\tau }\cdot \left( u_{f}(f_{stim}(t)) \right. \nonumber \\&\left. - [\beta + (1-\beta )u_{f}(f_{stim}(t))]\cdot a'(t)\right) \end{aligned}$$

31 $$\begin{aligned} a(t)&= g\cdot (a'(t)-a_0) \quad 0<a(t)\le 1 \end{aligned}$$

where *a*(*t*) is the activation and $a'(t)$ is the activation prior to scaling and thresholding by *g* and $a_0$, respectively. In this model, the time constant of isometric force development varies linearly with neural drive $u_f$ from $\tau$ when $u_f = 1$ (i.e.,maximum) to $\tau /\beta$ when $u_f = 0$ (i.e., no neural drive and the muscle is in relaxation). The gradation of the activation time constant allows the model to capture the increased speed of force rise during excitation compared to relaxation, which has been observed in muscle tissue (Zajac 1989).

The activation dynamics (Eqs. 30 and 31), force-frequency (Eq. 7), length-tension (Eqs. 15 and 16) and force-velocity (Eq. 21) models were implemented in MATLAB Simulink (variable step, auto selection of solver, 50 ms maximum time step) to simulate the muscle’s force generation in response to imposed length changes and neural excitation. The activation dynamics parameters of $a_0, g, \tau$ and $\beta$ were optimized to the data using MATLAB’s fminsearch by minimizing the mean squared error between the Simulink model response and the measured data.

#### I3 Model identification

**Fig. 3:**
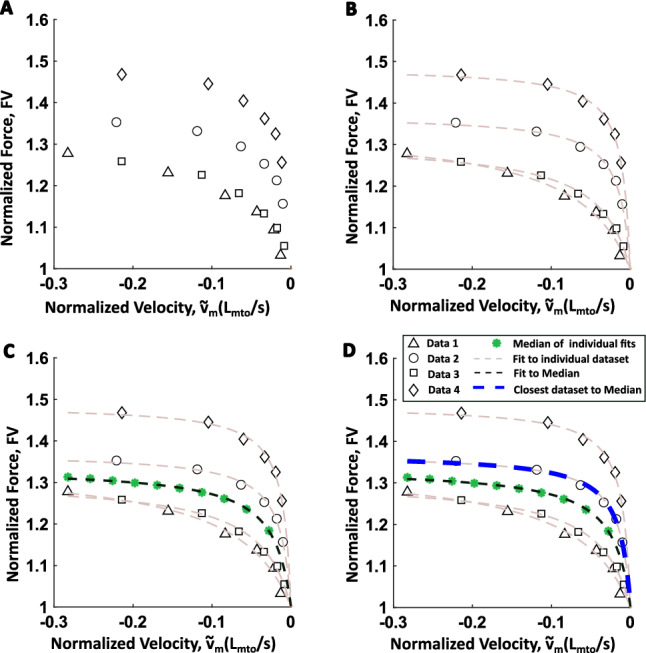
Due to variability between individuals, taking the median of all of the data may not accurately represent biologically plausible muscle dynamics. Therefore, the muscle model in this work was developed based on the individual animal whose muscle performed most closely to the median. The procedure for muscle model identification is illustrated here. **A** Raw data was collected from multiple muscles. Data from the lengthening force-velocity experiments is used here to demonstrate the procedure. **B** Data for each individual replicate was interpolated and fitted with the appropriate functional relationship (gray dashed line). **C** For the interpolated datapoints, the median $y_{med}$ is found (green dots) and a fit to the median found (black dashed line). **D** The experimental dataset which was closest to the fitted median was identified (blue-dashed line)

The inter-individual variability of biological system parameters may exhibit a large range of values. However, only specific sets of parameters produce functional behavior in a given individual (Loeb 2012; Golowasch et al. 2002; Blümel et al. 2012). As a consequence, using a measure of central tendency like the mean or median response may not correspond to a parameter space that results in functional behavior in nature. To capture the parameter space that is representative of the population and which was actually observed in the experiments, the individual animal data for each experimental type that was closest to the median of all animals was identified. The model fit for this individual was then implemented in MATLAB (Fig. 3D).

For the force-frequency dataset, the forces were measured at the same frequencies across all $n=5$ experiments. Hence, no interpolation between frequency values was needed. For the length-tension and force-velocity experiments however, the normalized lengths (${\tilde{l}}_m$) and velocities (${\tilde{v}}_{m}$), respectively, may not be the same across all experiments. To address this, the length-tension fit for a single length-tension experiment Eq. 8 was linearly interpolated between the minimum and maximum ${\tilde{l}}_m$ across all the data, at a spacing of 0.001. The force-velocity fit for a single force-velocity experiment Eq. 20 was linearly interpolated between the minimum and maximum ${\tilde{v}}_m$ across all the data, at a spacing of 0.0005 $L_{mto}/s$ (Fig. 3B).

The median value of the dependent variable, *y*, for all *n* experiments performed at the $i^{th}$ point of the equally spaced independent variable, *x*, is found at each *i* as:

32 $$\begin{aligned} y_{med,i} = median({\mathcal {L}}_{1}(x_{i},y_{1,i}),...,{\mathcal {L}}_{n}(x_{i},y_{n,i})) \end{aligned}$$

where ${\mathcal {L}}$ is the least squares fit (Fig. 3B). For force-frequency, length-tension and force-velocity datasets, ${\mathcal {L}}$ corresponds to equations (7, 8, 20), *x* corresponds to ( $f_{stim}$, ${\tilde{l}}_{m}$, ${\tilde{v}}_m$) and *y* corresponds to ($u_{f}$, *LT*, *FV*), respectively.

The least squares fit was then fit to $y_{med}$, resulting in ${\mathcal {L}}_{med}$ (Fig. 3C). The fit for an individual experiment (1, ..., *n*) which was closest to this median fit was found by finding the fit that had the lowest sum-of-squared error (*SSE*) to the median fit (Fig. 3D) as calculated by:

33 $$\begin{aligned} \begin{aligned} {\mathcal {L}}_{best} = min\{SSE\left( {\mathcal {L}}_{1}(x,y_1) - {\mathcal {L}}_{med}(x,y_{med})\right) ,... \\ SSE\left( {\mathcal {L}}_{n}(x,y_n) - {\mathcal {L}}_{med}(x,y_{med})\right) \} \end{aligned} \end{aligned}$$

### Model validation to *Aplysia* I1/I3 muscle electromyography data

To validate the I3 muscle model developed in this work, data relating stimulation frequency, muscle activity, and approximate force were collected from a semi-intact preparation of the buccal mass from a single adult *Aplysia* weighing 290 g. To collect this data, the buccal mass was removed from a fully anesthetized adult *Aplysia*. The halves of the I3 were separated and the odontophore was surgically removed. Subsequently, the I3 was sewn back together and the buccal mass was held in place at the bottom of a dish using SYLGARD 184 (Silicone Elastomer Kit, Dow Silicones Corporation, Midland, MI). The I1/I3 lumen was connected to a force transducer (GRASS force-displacement transducer FT03, Daytronic strain gage conditioner model 3170) using a needle and thread, an extracellular hook electrode was placed on the BN2, and an intracellular electrode (Huan et al. 2021) was applied to B3 for stimulation. B3 was stimulated at 20Hz with a pulse width of 40 ms for 5 s using an Arduino pulse generator. Electromyography (EMG) signals in the I1/I3 muscle complex were recorded via a hook electrode. All data were recorded using Axograph as described above.

The I1/I3 EMG signal was then rectified and normalized by the maximum EMG value. The envelope of the rectified and normalized EMG signal was found using MATLAB’s envelope() function (‘peak’ option, minimum separation of local minima of 1000 samples). The optimal scaling for the EMG to minimize the mean-squared error between the simulated and measured force response was found with MATLAB’s fminsearch function. The scaled envelope of the EMG can be considered the net neural input to the muscle (Zajac 1989), and was used as the $f_{stim}$ input to the force-frequency response $u_f$ in the activation dynamics (Eq. 30). The Simulink Hill-type I1/I3 model was used to simulate the force response to this rectified EMG envelope.

## Results

**Fig. 4:**
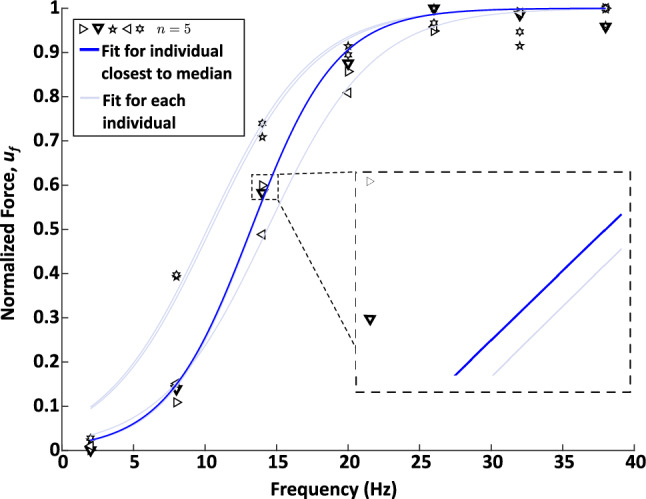
The force-frequency data shows a sigmoidal profile, with saturation of the force produced for frequencies $\ge$ 26 Hz. Markers represent the measured force response from $n=5$ individual *Aplysia*. Lighter lines represent sigmoidal fits to each individual animal. The response closest to the median is indicated by the heavier blue line and bolded symbol outline (downward-pointing triangle). The data for this animal was used for the force-frequency component of the Hill-type model. Note that the force-frequency data of 5 individuals is shown. The fits of the downward-pointing triangle (heavy blue line, closest to median), and the right-pointing triangle are extremely close. A zoomed-in view of the separation is shown in the inset

**Fig. 5:**
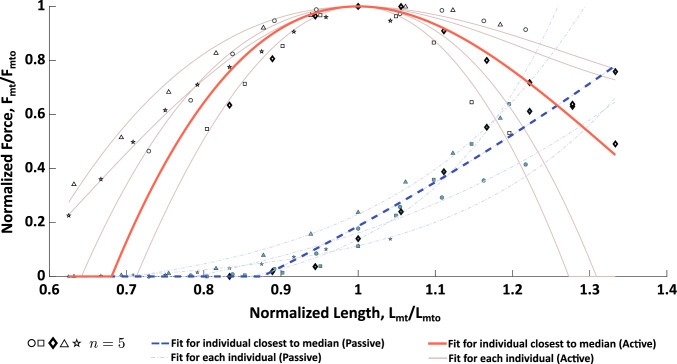
Active (open markers) and passive (filled markers) length-tension data. Markers represent the measured force response from $n=5$ individual *Aplysia*. Lighter lines represent length-tension fits to each individual animal. The response closest to the median is indicated by the heavier red line for the active length-tension, and by the heavier blue dashed line for the passive length-tension. The fit closest to the median for both active and passive length-tension curves both came from the same animal (diamond symbol). Hence, these fits were used for the active and passive length-tension properties of the Hill-type model. Note that despite the apparent linearity of the individual closest to the median’s passive length-tension curve, an exponential was fit. The passive length-tension data suggests that for normalized lengths smaller than $\sim 0.88L_{mto}$, the passive force is negligible. $L_0$, the rest length of the muscle, was found to be $\sim 0.89L_{mto}$

**Fig. 6:**
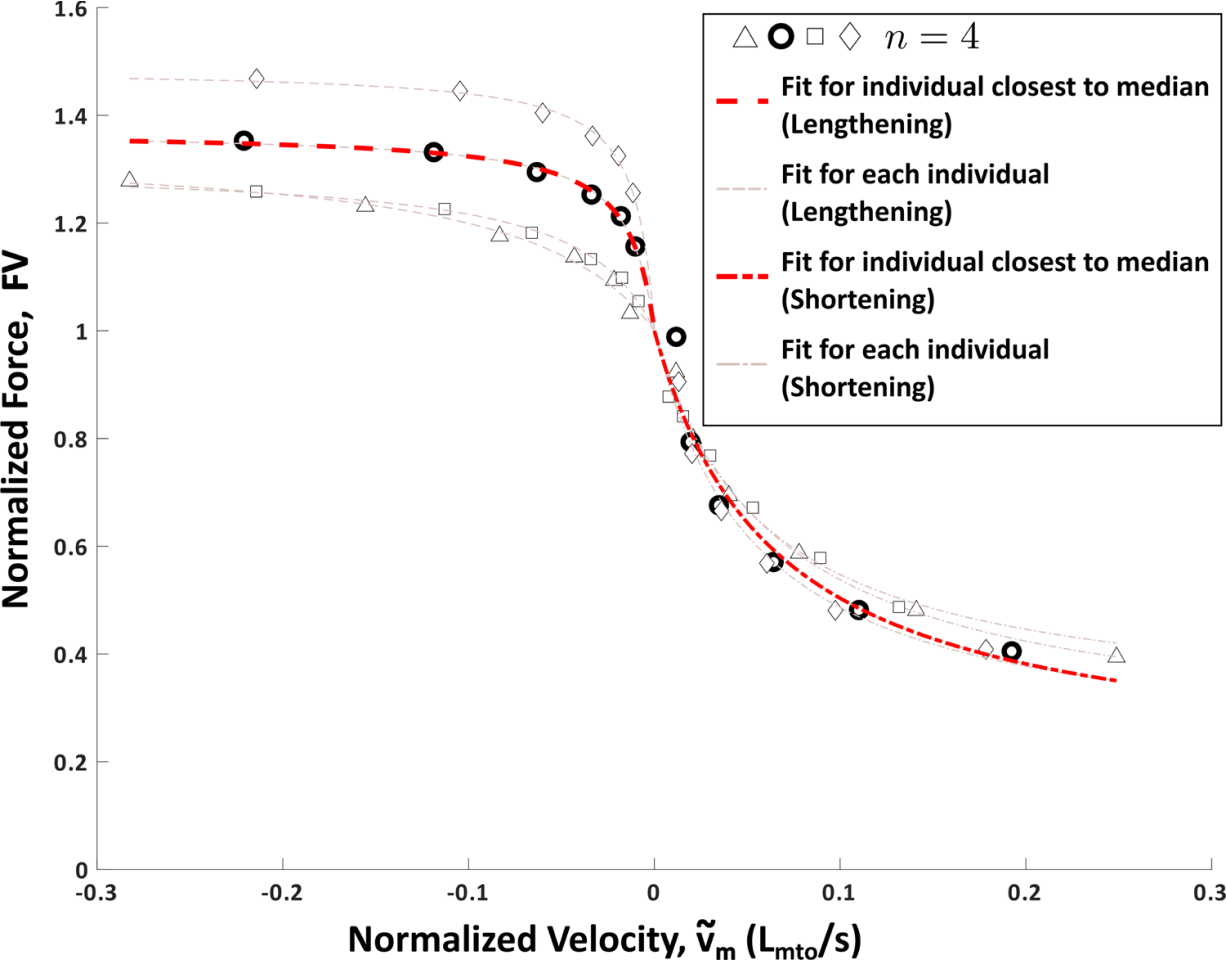
Force-velocity data for lengthening (negative velocities) and shortening (positive velocities). Markers represent the measured force response from $n=4$ individual *Aplysia*. Lighter lines represent the force-velocity model fit to each individual animal. The response closest to the median is indicated by the heavier red line and bolded symbols for lengthening and shortening. The fit closest to the median for both the lengthening and shortening force velocity responses came from the same animal (circle symbol). Hence, these fits were used for the force-velocity properties of the Hill-type model. Greater variability was observed in lengthening compared to shortening

**Fig. 7:**
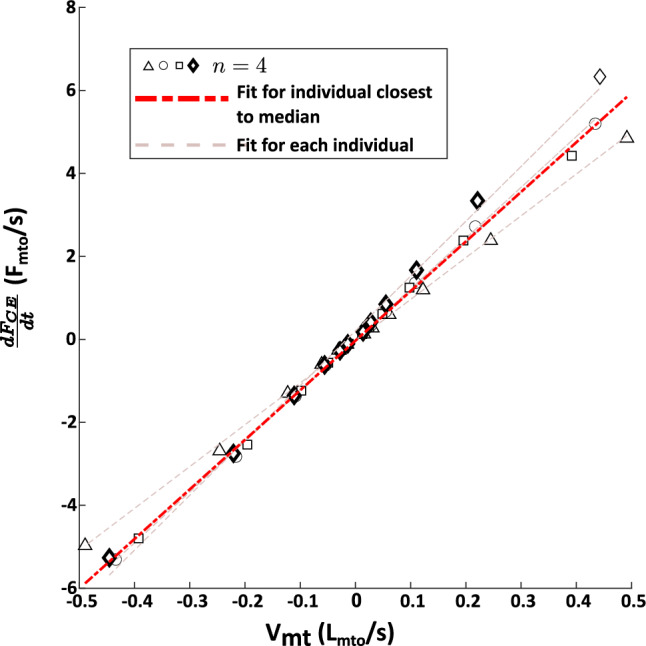
Estimate of series elasticity from the slope of the $\frac{dF_{CE}}{dt}$ vs $V_{mt}$ line. Markers represent the measured force response from $n=4$ individual *Aplysia*. Lighter lines represent linear fits to each individual animal. The response closest to the median (diamond symbol) is indicated by the heavier red line and bolded symbols. This fit was used for fitting the series elasticity in the Hill-type model

The results of the experiments and the full Hill-type model fit to the experimental data are presented below. Note that markers that are the same type between the force-frequency (Fig. 4), length-tension (Fig. 5), force-velocity (Fig. 6) and series elasticity (Fig. 7) data represent measured data from the same animal. The model parameters of the individual fits to each animal can be found in Table 2.

### Force and stimulus frequency relationship

During constant stimulation under isometric conditions, the normalized force was observed to rise with increasing stimulation frequency until reaching a plateau between 25-45 Hz (Fig. 4). Below 2 Hz stimulation, there was minimal increase in force (Fig. 4). There was no noticeable change in force at stimulation frequencies of 26 Hz, 32 Hz, or 38 Hz. Therefore 26 Hz or 32 Hz was used in all subsequent stimulation experiments.

### Length-tension properties of the I3 muscle

During isometric experiments to determine the normalized length-tension relationship of the I3 muscle, passive and active forces were measured and normalized to the optimal length found during each individual trial. The non-normalized peak active forces were found to vary amongst individuals from 1.33 N to 1.91 N (1.65 ± 0.22 N). Similarly, optimal muscle length varied from 16.31 mm to 24.03 mm (19.45 ± 2.95). For all muscles tested, active forces were measurable from 0.8 $L_{mto}$ to 1.2 $L_{mto}$, and several samples produced measurable forces even below 0.8$L_{mto}$ (Fig. 5). Passive forces were negligible prior to 0.87$L_{mto}$, after which passive force rose non-linearly (Fig. 5).

### Force-velocity properties of the I3 muscle

The relationship between force and velocity was measured using a series of lengthening and shortening experiments (Fig. 6). During shortening, the normalized force gradually decreased with increasing shortening velocity. In contrast, lengthening resulted in sharp increases in normalized force followed by plateauing with increasing lengthening velocity. Interestingly, higher variability was observed in the lengthening trials than in the shortening trials.

### Normalized parameter estimates for kinetic model

Using the experimentally measured force responses, normalized parameter estimates were performed to construct a kinetic model of I3 force production. First, the series elasticity was estimated based on the slope of the time derivative of the active force vs. muscle velocity data as detailed in Sect. 2.6.4 (Fig. 7). The length-tension relationship was modeled as a cubic fit using the median data in Fig. 5 as described in Sect. 2.6.2, resulting in the expressions:

34 $$\begin{aligned} f_{PE} = {\left\{ \begin{array}{ll} 0 &{}, {\tilde{l}}_{mt}<0.87l_{mto}\\ -1.89 + \\ 1.70\cdot e^{\left( 0.75{\tilde{l}}_{mt}-0.55\right) } &{} \text {, otherwise} \end{array}\right. }\nonumber \\ \end{aligned}$$

35 $$\begin{aligned} LT({\tilde{l}}_{mt}) = (7.44\cdot {\tilde{l}}_{mt}^3 - 29.74\cdot {\tilde{l}}_{mt}^2 + 37.17\cdot {\tilde{l}}_{mt} -13.87)\nonumber \\ \end{aligned}$$

To take into account the effect of the series elasticity, the active length-tension fit as a function of the contractile element length, ${\tilde{l}}_{m}$, is given by:

36 $$\begin{aligned} LT({\tilde{l}}_{m}) = (5.44\cdot {\tilde{l}}_{m}^3 - 21.15\cdot {\tilde{l}}_{m}^2 + 25.16\cdot {\tilde{l}}_{m} -8.49)\nonumber \\ \end{aligned}$$

Next, the force-velocity relationship was modeled as a double exponential function based on the median data in Fig. 6. This fit results in the relationship being modeled as:

37 $$\begin{aligned} {\tilde{v}}_{m} = -0.84\cdot e^{6.37\cdot (FV - 1.69)} + 1.36\cdot e^{-5.07\cdot FV} \end{aligned}$$

These equations are then combined with the force-frequency model, which was fit using least-squares and the median data in Fig. 4, resulting in the following force-frequency relationship:

38 $$\begin{aligned} u_{f}(f_{stim}) = \frac{1.55}{1.55+7.90\cdot e^{-0.33\cdot (f_{stim} - 8.33)}} \end{aligned}$$

Together Eqs. 34, 36, 37 and 38 combine to form the complete kinetic model for the I3 muscle. Using this kinetic model, the parameters of the activation dynamics were then estimated by the optimization procedure described in Sect. 2.6.5. These parameters are reported in Table 1.

**Table 1:** Parameter Estimates

Parameter	Value	Unit
$\tau$	0.60	s
$\beta$	0.15	
$a_0$	0.74	
*g*	3.82	
$L_{st}$	0	mm
$F_{mto}$	1.61	N
$L_{mto}$	18.03	mm
$L_{0}$	16.02	mm
$K_t$	11.95	

**Fig. 8:**
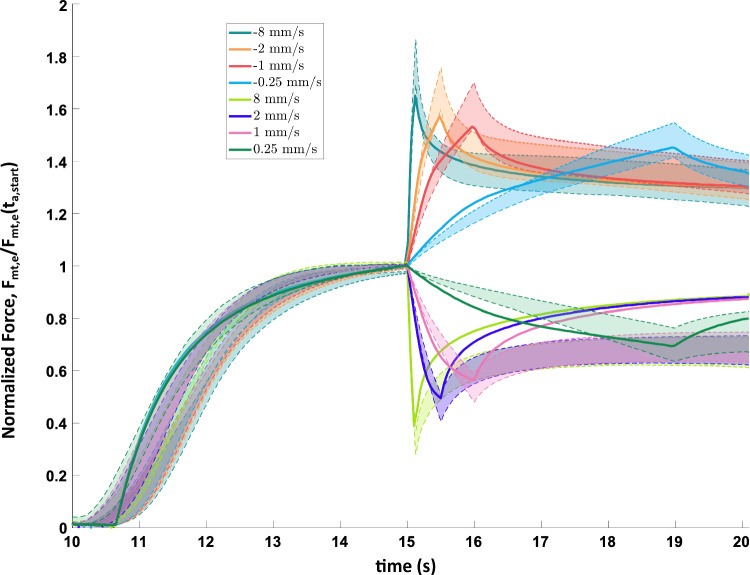
Comparison of predicted force response in simulated force-velocity experiments with the I1/I3 muscle model (solid lines) with experimental force-velocity results (shaded regions, mean ± 1 std. dev.). Forces were normalized by the isometric force prior to start of the ramp, $F_{mt,e}(t_{a,start})$ (see Fig. 2.C2). The model’s responses are qualitatively similar to the experimental results, though the model tends to under-predict the peak force change. This under-prediction of the force change could be due to the exclusion of the transient dynamic nature of the passive force change with a change in length from the model

### Force-velocity validation

The complete I1/I3 Hill-type muscle model was used to predict the force response from the imposed lengthening and shortening of the muscle during the force-velocity tests. During lengthening, the model captured the increase in contraction force with higher speeds (Fig. 8). During shortening, the model captured the decrease in contraction force with higher speeds (Fig. 8). After the ramp change in length had been completed, the model was able to predict the steady-state force for lengthening. For shortening, however, the steady-state reduction in force tended to be under-predicted by the model.

### Simulation of the I3 muscle response and comparison with experimental EMG data

**Fig. 9:**
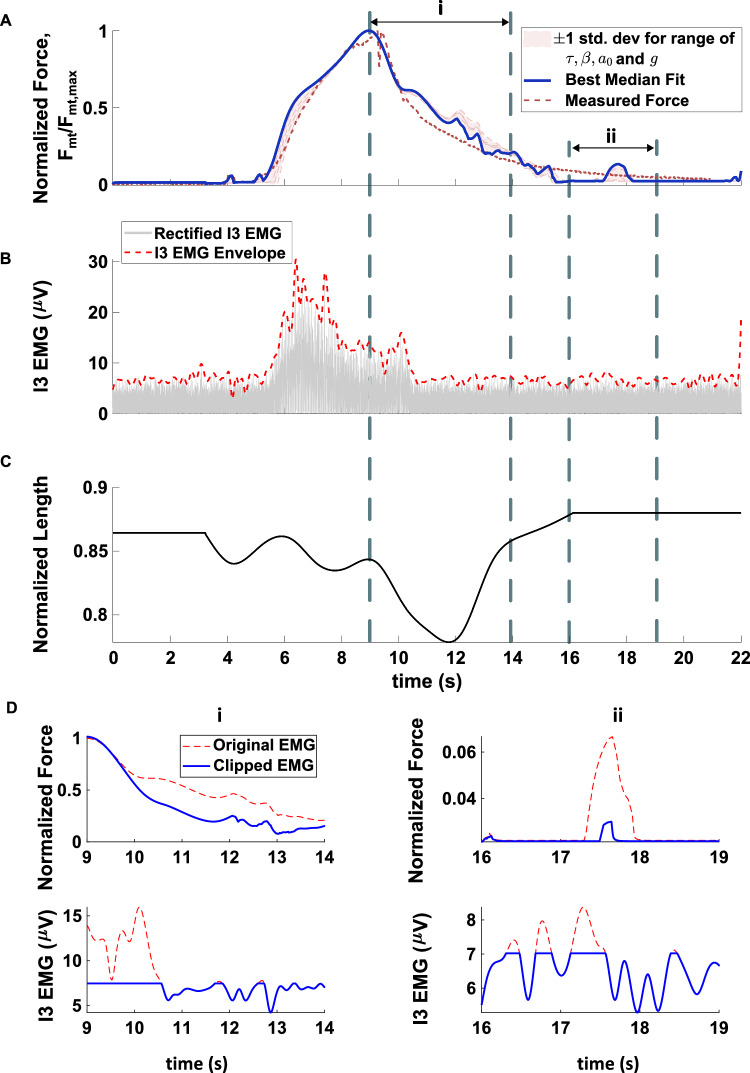
**A** Comparison of predicted normalized force response from the I3 model (solid blue line) with the $\tau$,$\beta$,$a_0$ and *g* from the best-fit force-frequency trial to the experimentally measured I3 force from B3 nerve stimulation (dashed line). The predictions of the model for the $\tau$, $\beta$, $a_0$ and *g* (see Table 2) of the other four force-frequency experiments are indicated in the shaded region. Data was normalized by the maximum force reached during the time evolution of the force response, $F_{mt,max}$. The inputs to the Simulink model were the rectified and scaled I3 EMG envelope (**B**) and normalized muscle length (**C**). The envelope was normalized by the maximum value ($\sim 30 \mu V$) and scaled (scale factor = 48.9). **D** Predicted effect on the force responses if the EMG input was clipped for periods *i* and *ii*. In period i, clipping the EMG reduced the predicted force slightly. In period *ii*, there were three successive peaks in the EMG input that were large enough to cause an increase in force. This was not observed in the measured data

To validate the I3 muscle model, the model was implemented in Simulink and compared to the experimentally obtained EMG data from measurements in I3 during B3 nerve stimulation. The predicted force response from the model was found to qualitatively match the measured force response (Fig. 9). Note that the measured response was advanced by 1.1 s to align with the predicted response. The parameter estimates for $\tau$, $\beta$, $a_0$ and *g* from the other four force-frequency experiments showed similar overall trends to the $\tau$, $\beta$, $a_0$ and *g* from the best-fit force-frequency data. The optimal EMG scaling for the best-fit force-frequency data was 48.9.

While the predicted force response from the model qualitatively matched the measured force response, there were discrepancies between the two. Notably, the simulated force response showed less filtering of the EMG input compared to the actual muscle. This resulted in greater sensitivity of the simulated force output to fluctuations in the EMG input (Fig. 9A-C). This effect was particularly noticeable during the decrease in force while the muscle shortened (period *i* in Fig. 9) and during the steady-state period where there was a small spike in the force even though there were no obvious fluctuations in either muscle length or the EMG (period *ii* in Fig. 9). In period *i*, clipping the EMG signal shows a reduction in the simulated force, but there are still subsequent small rapid changes in force that result from changes in the EMG (Fig. 9D i). In period *ii*, the increase in force in steady-state was caused by the presence of three peaks in the EMG envelope that were large enough to activate the muscle. When those peaks were clipped, the magnitude of that force spike was greatly reduced (Fig. 9D ii). No such spike was found during period *ii* for the real muscle, a possible indication of additional un-modelled filtering mechanisms within the muscle.

## Discussion

This work presents a Hill-type model of the *Aplysia* I1/I3 muscle complex based on experimental characterization of muscle mechanics, activation, and dynamics. The model was validated by comparison with experimental electromyography data recorded in a semi-intact preparation. This model can be used to improve biomechanical models of *Aplysia* feeding by providing details about I1/I3’s behavior. As seen in previous muscle characterizations of the I2 protractor muscle, our characterization of I1/I3 showed that while there is variability between individual animals, muscle behaviors for each animal fit the Hill-type model with a high degree of correlation.

### Physiological relevance of force-frequency behavior

The force-frequency data demonstrate that, for frequencies above 20 Hz, the magnitude of the force would saturate. Prior work has found that the B3, B6, and B9 neurons, which are the largest neurons that project on the BN2 and are primary drivers of I3 force generation, have median frequencies of 8.1 Hz, 9.9 Hz, and 10.1 Hz, respectively (Lu et al. 2015). The force-frequency data from this paper suggests that the force output of the I3 muscle would be relatively small at those frequencies. This agrees with prior observations that the separate firing of these neurons in biting-like patterns produced little force on their own (Lu et al. 2015). However, when those neurons fired simultaneously, either as a biting-like pattern before a swallowing-like pattern or as a swallowing-like pattern, there was a marked increase in the generated force of the I1/I3 muscle complex (Lu et al. 2015). This increase in force was greater than a linear summation of forces generated during separate firing of these neurons (Lu et al. 2015). From recordings of the *Aplysia* swallows where the B3, B6, and B9 neurons were treated as a single unit, the firing frequencies ranged from 15.66 to 42.27 Hz (Gill et al. 2020). This agrees with the force-frequency analysis reported here and may indicate that typical swallowing behaviors fall in the rising leg of the force-frequency curve, while maximal force can be achieved if needed *in vivo*.

### Kinematics of the I1/I3 muscle and relation to force-length and force-velocity behavior

Using the kinematics of the I1/I3 muscle complex that were measured by Neustadter et al. (2002), possible effects of the measured force-length and force-velocity curves on force generation of the I3 muscle during feeding *in vivo* can be determined. Based on the MRI images of the buccal mass during swallowing, the rest length of the I1/I3 muscle appears to occur at the end of the loss of the $\Gamma$ shape at which time the buccal mass is relaxing from peak retraction. This corresponds to the end of period T2 in Fig. 9 of Neustadter et al. (2002). Assuming that the dorsoventral length represents the diameter of the I1/I3 muscle with a uniform cylindrical cross-section, the $L_0$ is approximately 25 mm. The data presented in this work suggests that $L_0 = 0.89 L_{mto}$. This would imply that $L_{mto}$ for the data in Fig. 9 of Neustadter et al. (2002) is approximately 28 mm. Looking at the kinematic profiles of the I3 dorsoventral length during swallows implies that the I3 muscle primarily operates near the peak of the length-tension curve. At peak protraction, the length of I3 is approximately $1L_{mto}$. At this length, the I3 muscle produces its maximum active force, which facilitates the functional role as a retractor of the odontophore. At peak retraction, the length of the I1/I3 is approximately $0.9L_{mto}$, which allows the I3 to produce force at approximately 90% of the maximum value. Notably, at peak retraction, the I3 muscle is completing the shortening phase of the stretch-shorten cycle, and the passive length-tension properties are expected to be small. We estimate the passive force at peak retraction, just before the start of protraction of the odontophore, to be $\sim$4% of $F_{mto}$ based on the passive length-tension curve. This smaller amount of passive force during protraction in swallowing may help the I2’s role as an initiator of protraction because there would be less resistance to the forward movement of the odontophore from the I1/I3 muscle complex.

However, because the contraction of the I3 muscle happens primarily transverse to the direction of motion of the odontophore, it is also essential to understand the mechanical advantage of the I3 rings on the odontophore. Prior models of the posterior rings of I3 have shown that near peak protraction in swallowing, there is a context-dependent point where the I3 muscle switches function to that of a protractor (Novakovic et al. 2006). However, the model used by Novakovic et al. (2006) did not consider the effect of the more anterior rings of the I1/I3 muscle complex, which may have a mechanical advantage that facilitates retraction of the odontophore when it is near peak protraction in swallowing. The I1/I3 complex also exhibits spatial innervation, where the B38 neuron controls contraction of the anterior part of the I1/I3 near the jaw, which aids in clamping food during swallowing (McManus et al. 2014). In the work presented here, the effect of this spatial innervation was not investigated as the entire BN2 was stimulated. As a consequence, the estimates of $F_{mto}$ may represent an upper bound on the actual force the I1/I3 muscle complex could achieve. Moreover, from Fig. 9 of Neustadter et al. (2002), it is apparent that the dorsoventral length at the lateral groove is quite different from that at the jaws. This change in muscle length was not captured by the model presented in this work, which treated the entire I1/I3 muscle as a uniform cylinder. Hence, estimates of the force at the anterior portion of the I1/I3 muscle using $L_0 ~= 25 mm$ may under-predict the actual force.

The force-velocity properties may also influence the kinetic response of the muscle. From Neustadter et al. (2002), the maximum velocity of both lengthening and shortening during retraction is $\sim 0.27 l_{mto}/s$. From the characterization of I1/I3 in the present work (Fig. 6), the active damping for both lengthening and shortening at these velocities would tend to resist rapid perturbations. These damping properties may play an important role in *Aplysia* feeding since when the animal is underwater, the relaxation time constant of muscle activation of the I1/I3 is large relative to the activation time constant and may limit how quickly cyclic feeding behavior can be initiated. Because of this limitation in how quickly *Aplysia* may react to disturbances, these active damping properties may stabilize feeding behavior without the need for closed-loop reorganization of the neural commands sent to the periphery to counteract these short-term disturbances. However, for disturbances over longer timespans (100 s of milliseconds), such as when feeding on unbreakable seaweed, *Aplysia* can alter the duration and frequency of motor output of the nervous system to increase the amount of force generated during retraction (Gill et al. 2020).

### Model response in comparison to *in vivo* neural commands and behavior

A notable characteristic of smooth muscle is its ability to efficiently sustain contraction, which often comes at the expense of speed (Sulbarán et al. 2015; Butler and Siegman 2010). This contrasts with striated muscles, which are frequently used to effect rapid movements (Sulbarán et al. 2015). The I3 muscle, with its similarity to smooth muscle in vertebrates and invertebrates (Lu et al. 2015), likewise exhibits a slower production of force in response to neural excitation when compared to many vertebrate striated muscles. For instance, based on Eq. 30, the time constant at maximum muscle excitation is $\tau = 0.60$ s (Table 1) (Zajac 1989). The time constant during relaxation (i.e., when the muscle is deactivated, $u'(t)=0$) is $\tau _{relax} = \tau /\beta = 4.00$ s. In comparison, vertebrate muscle typically have $\tau$ in the range of 12 - 20 ms, and $\tau _{relax}$ in the range of 24-200 ms (Pandy 2001).

The activation and relaxation time of the I3 muscle may play an important role in the feeding behavior of *Aplysia*. For instance, prior studies of the closer and opener muscles of *Aplysia*’s radula revealed that without modulation of the intensity of contraction and relaxation rate of those muscles, there would be insufficient time for the opener and closer muscles to relax before the next contraction, which would result in failed biting behavior (Weiss et al. 1992). While larger time constants for muscle relaxation than excitation are a well-known feature (Zajac 1989), relative to the I2 muscle, the increase in the relaxation time constant is noteworthy. While the I3 muscle has a smaller time constant of activation relative to the I2 (0.60 compared to 2.45s (Yu et al. 1999), $p<0.05$, see Table 3), the I3 muscle’s relaxation time constant is comparable to I2’s (4.00s compared to 3.49s, $p>0.05$, see Table 3). This implies that while the I3 muscle can generate force more quickly than the I2, it takes longer for the generated force to decay. This may have implications for the timing of neural activity during *Aplysia* feeding behavior. For instance, in swallowing, there is a noticeable period of quiescence from the end of B3/B6/B9 firing during retraction of the buccal mass to the start of B31/B32 firing during protraction (Webster-Wood et al. 2020; Lu et al. 2015). This delay may be necessary to allow the force in the I3 muscle to decay sufficiently for the next phase of feeding motion to occur. The longer relaxation time may also facilitate more efficient generation of forces during swallowing by reducing the amount of time neural excitation is required.

### Comparison to the I2 muscle

In addition to differences in the time constants for activation and relaxation between the I2 and I1/I3 muscle, there were also other notable differences in the mechanical properties and force generation capabilities of the two muscles. The I1/I3 was found to have a much higher peak isometric force $F_{mto}$ (1.61 N compared to 0.15 N, $p<0.05$, see Table 3) and series elastic stiffness $K_t$ (11.95 compared to 5 $F_{mto}/L_{mto}$ (Yu et al. 1999), $p<0.05$, see Table 3). This may reflect the fact that the I1/I3 muscle complex is much thicker and may have a larger composition of cartilage (Lu et al. 2015; McManus et al. 2014) and other passive structural materials than the I2 muscle.

### History-dependent effects

Given the size of the I1/I3 complex, it is possible that diffusion is insufficient to bring glucose and oxygen to the entirety of the muscle during isolated testing. Indeed, it was observed that the longer the muscle was maintained in the experimental testing apparatus, the lower the force response became. A decrease in muscle force production after repeated stimulation over a long time period is not unexpected in isolated muscle, as such preparations often establish diffusion gradients of necessary metabolic substances across the muscle (Allen et al. 2008). Such diffusion gradients can lead to the development of an anoxic core in the muscle that can result in fatigue, which may not reflect physiological sources of fatigue *in vivo*. To capture the effect of fatigue, future work may augment this Hill-type muscle to incorporate phosphate kinetics into the activation dynamics (Rockenfeller et al. 2020), or adding an experimentally determined “fatigue function" in the activation (Tang et al. 2007).

Beyond fatigue, other history-dependent effects that were not captured by this version of the Hill-type model could have an effect on the observed force production trends. For instance, it was observed that the model tended to under-predict the drop in force at steady-state after shortening (Fig. 8). This inability to capture force depression during shortening with the Hill-type model has been shown in prior studies in skeletal muscle (McGowan et al. 2010), and was also observed in the I2 muscle of *Aplysia* (Yu et al. 1999). The muscle also exhibited passive forces which decayed slowly with time after a change in length. These dynamic changes in passive force were not included in this model but can be included in future work to further improve fit to animal data. To better account for such force depression, future work can look at augmenting the Hill-type model of the I3 to incorporate the effect of the net work done by the muscle (McGowan et al. 2010).

### Limitations

There are several limitations of this model that should be discussed. First, this model was fit based on adult *Aplysia* within a specific weight range (200-450 g). Muscle properties likely vary as a function of animal age, sexual maturity, and overall size (Rogers et al. 2024). This should be investigated in future studies. Second, given the variability observed between animals, the method of fitting the model to the median individual in this work may be limited by the sample size in this study. Future work focused on modeling a specific individual will need to investigate how to shape this model to the particular individual based on external kinematic measures such as retraction duration and swallowing force. The Hill-type model of the *Aplysia* I1/I3 complex found in this work includes active damping in the form of the force-velocity properties, but it does not incorporate the dynamic nature of the passive forces. Observations of force response during the length-tension experiments after a length change and from the force-velocity experiments indicate that passive viscoelasticity exists in the I1/I3 muscle, and the transient passive forces can be as large as $\sim$350 mN. Because of this transient effect, a rest period of 180 s between each length-tension length change was necessary to allow such passive forces to begin to converge. However, as passive muscle forces often follow a power-law decline, the passive force at 180 s may have still decreased further if given a longer rest period to converge before stimulating the muscle (von Twickel et al. 2019). While a longer rest period would allow us to approach the true steady-state passive force, if the rest period was too long, the muscle would deteriorate. Future work may look at incorporating an additional passive damping element in the Hill-type model to capture this behavior.

**Table 2:** Parameters of the models fitted for each animal. Parameters representing the best median fit are highlighted in **bold**

Animal	Symbol	Force-Frequency,	Passive *LT*,	Active $LT(l_{mt})$,	Active $LT(l_{m})$,	IFV,	FV,
		${\textbf{A}}=\langle A_1,A_2,A_3,A_4\rangle$	${\textbf{C}}=\langle C_1,C_2,C_3,C_4\rangle$	${\textbf{B}}=\langle B_1,B_2,B_3,B_4 \rangle$	${\textbf{Y}}=\langle Y_1,Y_2,Y_3,Y_4 \rangle$	${\textbf{E}}=\langle E_1,E_2,E_3,E_4,E_5 \rangle$	${\textbf{D}}=\langle D_1,D_2,D_3,D_4 \rangle$
1	$\triangleright$	[1.54, 7.97, 0.34, 8.40]					
2	⁎	[2.90, 6.63, 0.27, 7.11]					
3	$\diamond$		**[-1.89, 1.70, 0.75, 0.55]**	**[7.44, -29.74, 37.17, -13.87]**	**[5.44, -21.15, 25.16, -8.49]**	[1.17, 13.52, 1.60, 15.36, -10.78]	[-0.79, 0.054, 0.49, 0.01]
4	$\triangle$		[-0.10, 1.01, 4.11, 5.23]	[4.33, -16.54, 20.07, -6.87]	[8.35, -27.35, 28.94, -8.98]	**[0.99, 13.03, 1.37, 2.32, -5.71]**	**[-0.77, 0.07, 0.35, 0.07]**
5	$\circ$		[-0.25, 1.01, 2.33, 3.21]	[7.71, -28.16, 33.18, -11.73]	[9.88, -33.87, 37.20, -12.26]	[0.86, 15.37, 1.45, 8.16, -9.14]	[-0.82, 0.07, 0.37, 0.01]
6	$\square$		[-0.11, 0.64, 5.80, 6.74]	[-2.35, -5.74, 18.53, -9.44]	[20.09, -69.39, 76.57, -26.35]	[0.90, 13.74, 1.37, 2.83, -6.19]	[-0.71, 0.06, 0.31, 0.04]
7	$\triangleleft$	[0.93, 7.06, 0.27, 6.83]					
8	$\triangledown$	**[1.55, 7.90, 0.33, 8.33]**					
9	$\star$	[2.82, 6.70, 0.27, 7.17]	[-0.03, 1.32, 4.69, 6.90]	[-7.32, 13.72, -5.49, 0.087]	[-9.13, 14.42, -3.41, -0.95]		

Animal	Symbol	$F_{mto}$ (N)	$L_{mto}$(mm)	$L_0$(mm)	$K_t$	$\beta , \tau , a_0, g$	
1	$\triangleright$					0.13, 0.66, 0.74, 3.82	
2	⁎					0.17, 0.55, 0.57, 2.33	
3	$\diamond$	**1**.**61**	**18**.**03**	**16**.**02**	**11**.**95**		
4	$\triangle$	1.62	16.31	11.31	10.08		
5	$\circ$	1.76	18.46	14.45	12.25		
6	$\square$	1.91	20.44	17.44	13.21		
7	$\triangleleft$					0.12, 0.70, 0.76, 4.17	
8	$\triangledown$					**0.15, 0.60, 0.74, 3.85**	
9	$\star$	1.33	24.03	16.02		0.13, 0.70, 0.66, 2.94

**Table 3:** One-sample t-test to compare model parameters from the I3 data (see Table 2) to the values reported in Yu et al. (1999) for the I2 muscle

Parameter	I3 mean ± 1 std. dev (num. samples)	I2 value (Yu et al. 1999)	*p* (1 sample t-test)
$F_{mto}$(N)	1.65±0.21 (5)	0.15	$<0.0001$
$K_t$	11.87±1.31 (4)	5	0.0019
$\tau$ (s)	0.64±0.07 (5)	2.45	$<0.0001$
$\frac{\tau }{\beta }$(s)	4.62±1.04 (5)	3.48	0.071

Though the model produced a good qualitative match to the measured force with the rectified EMG envelope the predicted response was observed to lead the measured response by 1.1 s. The exact reason for this additional delay in the EMG validation is not entirely clear but may be related to the nonlinear manner in which motor neuron firing is transformed to muscle contraction (Brezina and Weiss 2000). Prior studies have shown that the properties of this neuromuscular transform (NMT) in *Aplysia*, such as the kinetics and amplitude of contraction, can be tuned via neurotransmitter-mediated modulation (Brezina et al. 2000; Fox and Lloyd 1997). Fox and Lloyd showed that the presence of serotonin can result in a decrease in the latency between B3 and B38 firing and the onset of contraction in the anterior portion of the I3 (Fox and Lloyd 1997). This decrease in latency can be on the order of seconds (Fox and Lloyd 1997). Such neurotransmitter-mediated changes to the activation dynamics were not captured in this model.

These changes to the activation dynamics could also help to bring the predicted force response to the EMG envelope closer to the measured response. For instance, the measured force showed a greater ability to filter rapid changes to muscle length and the EMG than the simulated Hill-type model did. This is exhibited in the descent phase of the force in Fig. 9A (time period *i*), and in steady-state where neither the EMG nor the length of the muscle was changing yet the model predicted a small rise and subsequent decay in the force produced by the muscle (time period *ii*). The measured force response from the real animal did not show such sensitivity to changes in the EMG. These neurotransmitter-mediated changes to the activation dynamics could potentially introduce additional filtering and delays to better match the measured force response. Future work could extend the current model to include such neurotransmitter-mediated changes to the NMT, which may facilitate a wider range of *Aplysia* feeding behavior than if the NMT was fixed (Brezina et al. 2000).

## Conclusions

This work expands the understanding of the feeding apparatus in *Aplysia californica* by creating and validating a Hill-type model of the I1/I3 retractor muscle complex. This muscle system is critical in feeding behavior, playing a major role in all three key multifunctional behaviors, biting, swallowing and rejection. Although the behavior of the muscle was found to vary somewhat between individuals, by fitting the model to the individual closest to the experimental data median, this model approximates I1/I3 contractile dynamics in adult *Aplysia*. Future work should assess how muscle properties vary with animal age, given the large changes in the size of the buccal mass during *Aplysia* development . This model will allow future biomechanical simulations to better capture the dynamics of the periphery during *Aplysia* feeding to investigate how the neuromuscular system coordinates and controls complex multifunctional behavior.

## Appendix A, A.1 Individual animal fits

The model parameters for each individual animal are summarized in Table 2.

## Appendix A, A.2 I2 vs. I3 model parameters comparison

To determine whether $F_{mto}$, $K_t$, $\tau$ and $\tau /\beta$ were significantly different for the I3 compared to the I2, a one-sample t-test was used. It was found that $F_{mto}$, $K_t$ and $\tau$ were significantly different for the I3 measured values compared to the reported I2 values (Table 1 in Yu et al. (1999)) at the $5\%$ significance level (Table 3). However, the relaxation time constant, $\tau /\beta$, was found to not be significantly different.
